# A multicenter, hospital-based and non-inferiority study for diagnostic efficacy of automated whole breast ultrasound for breast cancer in China

**DOI:** 10.1038/s41598-021-93350-1

**Published:** 2021-07-06

**Authors:** Yujing Xin, Xinyuan Zhang, Yi Yang, Yi Chen, Yanan Wang, Xiang Zhou, Youlin Qiao

**Affiliations:** 1 Department of Interventional Therapy, National Cancer Center/National Clinical Research Center for Cancer/Cancer Hospital, Chinese Academy of Medical Sciences and Peking Union Medical College, Beijing, 100021 China; 2 Department of Interventional Radiology, First Hospital of Shanxi Medical University, Taiyuan, 030001 Shanxi China; 3 Department of Epidemiology, National Cancer Center/National Clinical Research Center for Cancer/Cancer Hospital, Chinese Academy of Medical Sciences and Peking Union Medical College, Beijing, 100021 China

**Keywords:** Cancer imaging, Cancer screening, Cancer

## Abstract

This study is the first multi-center non-inferiority study that aims to critically evaluate the effectiveness of HHUS/ABUS in China breast cancer detection. This was a multicenter hospital-based study. Five hospitals participated in this study. Women (30–69 years old) with defined criteria were invited for breast examination by HHUS, ABUS or/and mammography. For BI-RADS category 3, an additional magnetic resonance imaging (MRI) test was provided to distinguish the true negative results from false negative results. For women classified as BI-RADS category 4 or 5, either core aspiration biopsy or surgical biopsy was done to confirm the diagnosis. Between February 2016 and March 2017, 2844 women signed the informed consent form, and 1947 of them involved in final analysis (680 were 30 to 39 years old, 1267 were 40 to 69 years old).For all participants, ABUS sensitivity (91.81%) compared with HHUS sensitivity (94.70%) with non-inferior Z tests, *P* = 0.015. In the 40–69 age group, non-inferior Z tests showed that ABUS sensitivity (93.01%) was non-inferior to MG sensitivity (86.02%) with *P* < 0.001 and HHUS sensitivity (95.44%) was non-inferior to MG sensitivity (86.02%) with *P* < 0.001. Sensitivity of ABUS and HHUS are all superior to that of MG with *P* < 0.001 by superior test.For all participants, ABUS specificity (92.89%) was non-inferior to HHUS specificity (89.36%) with *P* < 0.001. Superiority test show that specificity of ABUS was superior to that of HHUS with *P* < 0.001. In the 40–69 age group, ABUS specificity (92.86%) was non-inferior to MG specificity (91.68%) with *P* < 0.001 and HHUS specificity (89.55%) was non-inferior to MG specificity (91.68%) with *P* < 0.001. ABUS is not superior to MG with *P* = 0.114 by superior test. The sensitivity of ABUS/HHUS is superior to that of MG. The specificity of ABUS/HHUS is non-inferior to that of MG. In China, for an experienced US radiologist, both HHUS and ABUS have better diagnostic efficacy than MG in symptomatic individuals.

## Introduction

Breast cancer is the most common cancer in women worldwide^1^. Over half of all cases (53.0%) occur in less developed regions of the world^1,2^. There is a trade-off between benefit and harm of breast cancer screening^1,3–9^. False-positive results lead to anxiety and unnecessary, often invasive diagnostic procedures. Breast cancer screening can often over-diagnose disease and lead to unwarranted treatment. The accuracy of screening services may vary from one population to another, implying that a single screening procedure may not be universally effective.

Chinese government has launched a national breast cancer screening program in 2009, but has not yet formed a reliable breast cancer screening program that is suitable for national and regional population characteristics. Although mammography has been shown to reduce mortality in breast cancer, its accuracy is lower in women with high-density breast tissue and in young women in particular. In addition, mammography equipment is not affordable in rural areas in China. Furthermore, Asian women characteristically have higher-density breasts than women from other ethnic groups^10,11^. Irrespective of the ethnic origin, about 60% of women in their 40s are estimated to have dense breasts^12–14^. Given this, ultrasonography could offer a low-cost way to improve sensitivity and detection rates of early cancers in women with dense breasts^7^. It is imperative to establish a suitable breast cancer screening program in China that would be cost-effective and would improve screening benefits. Therefore, it is crucial to conduct an evidence-based study for the effectiveness of ultrasound techniques for the detection of breast cancer in the female population in China.

Although handheld ultrasound (HHUS) can be used to screen the whole breast, the technique is time-consuming and is less likely to achieve a standardized image because of the mobility of the breast tissue and the high degree of operator-dependence. This has become a major obstacle to the acceptance of ultrasound as a breast cancer screening technology in poor areas of developing and middle-income countries. Currently, breast ultrasonography can be performed using equipment for automated breast ultrasound systems (ABUS) in which all the breast tissue can be covered in a reproducible manner^18–21^. The anticipated advantage of ABUS systems is the decoupling of image acquisition and reading, which improves the possibilities for implementing breast ultrasonography in a screening setting. ABUS reduces the operator's dependency and can obtain series of reproducible standardized breast ultrasound images^22–28^.

To obtain reliable clinical epidemiological evidence that US can become a primary screening tool, long-term studies in large randomized controlled trials are needed. The time cost of such studies and the risk of negative results are high. To this end, we first design a non-inferior diagnostic efficiency study that takes less time and requires less samples. Its purpose is to provide initial evidence for future large randomized cohort studies. We have designed this multicenter hospital-based study to determine the efficacy of ABUS. Our goal was to compare ABUS, X-ray and HHUS and to determine if ABUS could be a suitable methodology for breast cancer screening.

## Methods

### Study protocol

**Figure 1:**
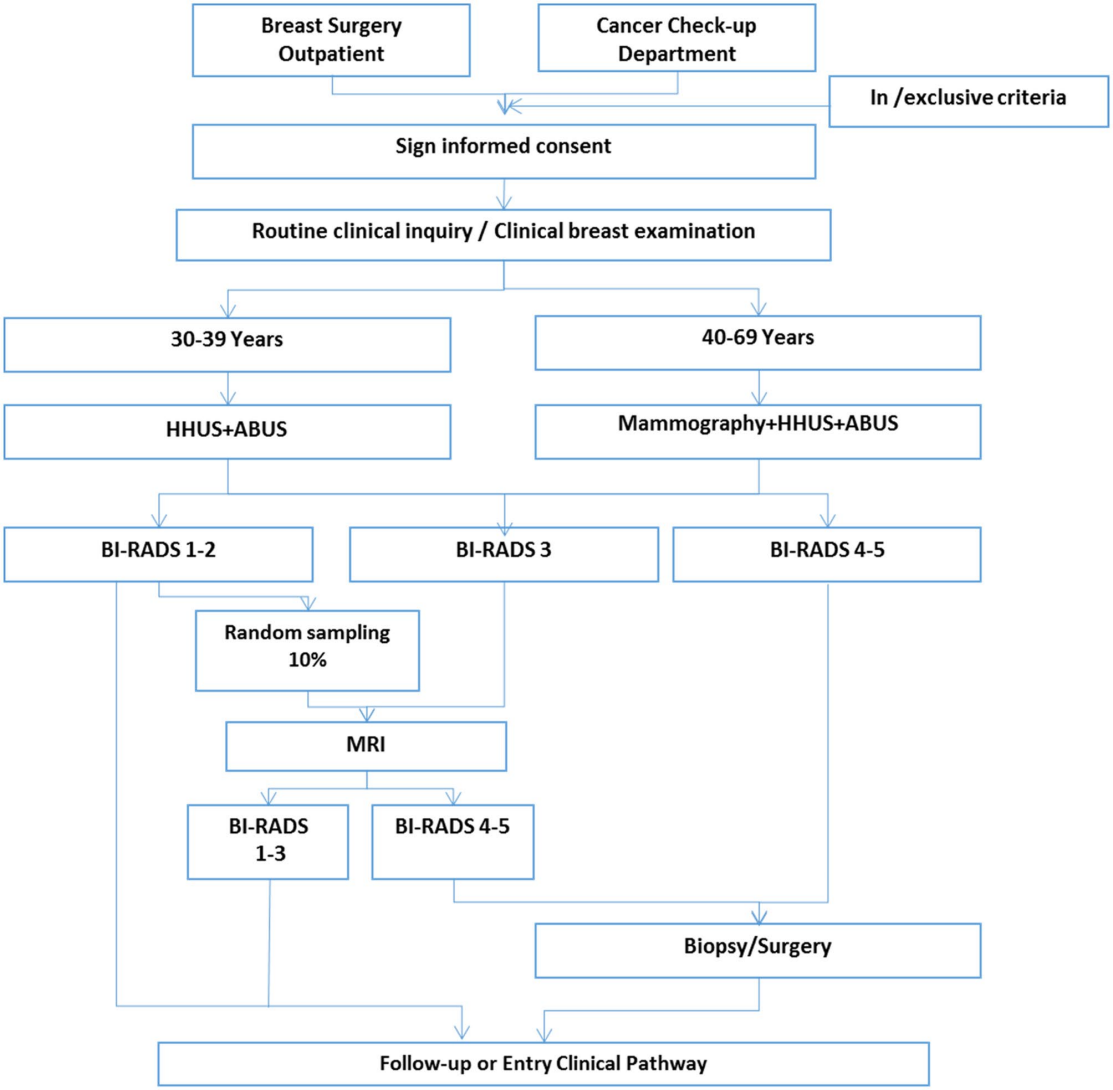
Flowchart showing patient selection and study design.

This STUDY was a hospital-based, multicenter, non-inferiority clinical trial that compared the diagnostic performance of ABUS, HHUS and mammography in Chinese women. In short, main procedures of this study were shown in Fig. 1. All women from outpatient were invited to participant in our study, eligible participants signed the informed consent form were enrolled, and a face-to-face questionnaire interview including social-demographic and potential breast cancer risk factors information was conducted by trained health workers. Then all enrolled participants underwent both HHUS and ABUS examination, successively. Women aged between 40 and 69 years old received an extra MG test, but the younger female group did not due to radiation. The study was approved by the Institutional Review Board of Cancer Institute, Chinese Academy of Medical Sciences (IRB approval No.15-061/988) and the Institutional Review Board of all participating hospitals. Informed consent was obtained from all participants for this prospective analysis. All the procedures above strictly followed clinical routines and guidelines.

### Inclusion criteria

Inclusion criteria were (1) female patients 30–69 years old; (2) woman who visited doctor for breast cancer examination and (3) no visible signs of breast cancer. Exclusion criteria were: (1) women who were pregnant, breastfeeding or planning to become pregnant; (2) lumpectomy history, contralateral mastectomy, breast augmentation; (3) surgical or percutaneous biopsy in the last 12 months; (4) diagnosis or treatment for cancer in the last 12 months. In each hospital, at least 300 subjects were scanned following similar routine and workflow (Fig. 1).

### Qualification of research center and staff

A total of five hospitals were included in this study. They are: Sun Yat-sen University Cancer Hospital; Chinese Academy of Medical Sciences Cancer Hospital; Tianjin Medical University Affiliated Tumor Hospital; Hangzhou First People's Hospital; Shanghai Jiaotong University Affiliated Xinhua Hospital.

The system under evaluation in this study was the Invenia 3D-Automated Breast Ultrasound System (ABUS), manufactured by GE Healthcare (Sunnyvale, CA USA). ABUS is a computer-based system for evaluating the complete breast. For each evaluation, each breast was imaged in three views: lateral (LAT), anteroposterior (AP) and medial (MED) with an automated 6 to 14 MHz linear array transducer attached to a rigid compression plate (covering areas of 15.4 × 17.0 × 5.0 cm). Each view acquired up to about 300 2D images and reconstructed in the coronal plane from the skin to the chest wall. The standardized review process involves using a patented, thick-slice coronal plane for quick navigation through the breast, as well the use of “survey mode,” which is similar to cine and allows radiologist rapid interpret of many images. The acquisition time for each view was approximately 60 s, with about 3–4 min per breast.

HHUS was performed in the supine position by experienced radiologists. The devices used to conduct HHUS included the GE LOGIQ9 (GE Medical Systems, Milwaukee, WI, USA), the Aixplorer system (Supersonic Imagine, Aix en Provence, France), the iU22 Ultrasound System (Philips Medical Systems, Bothell, WA, USA) and the s2000 (Siemens Medical Solutions, Mountain View, CA, USA).

The devices used to perform mammography and obtain mammographic images included the GE Sengraphe DS (GE Medical Systems, Milwaukee, WI, USA), the Hologic Selenia (Hologic, Bedford, MA, USA) and Fujifilm FDR MS-2500 (Fujifilm Corp, Tokyo, Japan). Generally, HHUS was performed by 10 radiologists, all with ≥ 5 years of experience in US examination and diagnosis. ABUS diagnosis was made also by 5 radiologist with at least 5 years’ experience of HHUS. MG diagnosis was performed by another 10 radiologist , all with at least 5 years of experience in MG diagnosis. MRI diagnosis was performed by the other 5 radiologist, all with ≥ 5 years’ experience in MRI diagnosis of breast. The radiologist performing and interpreting the US images and a different radiologist interpreting the MG were not permitted to know the results of the other current screening examination until their interpretations had been recorded, although prior breast imaging (if any) was available together with risk factor and biopsy/surgical history.

BI-RADS assessment results were sorted into six categories: 0 = incomplete, 1 = normal, 2 = benign, 3 = probably benign, 4 = suspicious, 5 = highly suggestive of malignancy, for HHUS, ABUS, and MG. The highest BI-RADS classified result among HHUS, ABUS, and MG would be the referral reference. For BI-RADS category 3, a magnetic resonance imaging (MRI) test was necessarily provided to distinguish the true negative results with false negative results. For BI-RADS category 1 to 2, there was no referral expecting that 10% of them were randomly selected to do MRI examination. For women classified as category 4 or 5, either core aspiration biopsy or surgical biopsy was done and a pathological diagnosis was followed. The MRI BI-RADS were also assessed. Women with BI-RADS category 1 to 3 of MRI would be considered to be negative. Otherwise, the woman would receive a biopsy examination to get the pathological information.

### Statistical analysis

Sensitivities and specificities among three methods (ABUS, HHUS and MG) were compared by non-inferior Z tests (non-inferior value was 0.06) by the score method (35–37), in which the variance was calculated by the restricted maximum likelihood estimation to estimate proposed by Nam (37). When the non-inferior test achieved P less than 0.025 and the test subjects get higher sensitivity or specificity estimates, the further superior test (the superior value was set to be 0) was conducted using Mcnemar. The sensitivity, specificity, false positive rate (FPR) and accuracy (AC) were calculated. Cancer size and number distribution was recorded.

### Sample size determination

The sample size was determined based on the need for a sufficient number of women with breast cancer to adequately evaluate the performance of ABUS, HHUS, and MG. According to American College of Radiology Breast Imaging Reporting and Data System (BI-RADS) distribution, the particular sample size is allocated into different BI-RADS categories: 40% in the BI-RADS 1–2 category, 35% BI-RADS 3, 12.5% BI-RADS 4 and 12.5% BI-RADS 5. All women were invited for breast cancer service MG, HHUS and ABUS from February 2016 to March 2017. For the BI-RADS category 3, a magnetic resonance imaging (MRI) test was provided to distinguish between negative results and false negative results.

## Results

From February 2016 to March 2017, a total of 2844 women consented to participate in our study. 1947 women were eligible for the study and completed scanning examination. Breast density was also reassessed by the radiologist and classified by using BI-RADS density category 1 (“almost entirely fat”), category 2 (“scattered fibroglandular densities”), category 3 (“heterogeneously dense”), or category 4 (“extremely dense”).” 24.31% of women were classified as having BI-RADS breast density type1-2, 75.69% type 3–4 by radiologists (Table 1). For analyses, category 1-2was categorized as “low-density breasts,” and categories 3–4 were defined as high-density breasts.

**Table 1:** Patient demographic and clinical characteristics at enrollment.

Characteristic	Total (n = %)	30–39 age group	40–69 age group
Age(y, mean ± SD)	45.40 (9.77)	35.11 (2.93)	50.92 (7.40)
Weight			
≤ 50 kg	329 (16.90)	155 (22.79)	174 (13.73)
51–60 kg	927 (47.61)	337 (49.56)	590 (46.57)
61–70 kg	515 (26.45)	141 (20.74)	374 (29.52)
> 70 kg	176 (9.04)	47 (6.91)	129 (10.18)
Menopausal status			
No. of premenopausal women	1399 (71.85)	675 (99.26)	724 (57.14)
No. of postmenopausal women	548 (28.15)	5 (0.74)	543 (42.86)
No. with unknown status	0 (0)	0 (0)	0 (0)
BI-RADS			
Type 1	334 (17.15)	115 (34.43)	219 (65.57)
Type 2	452 (23.22)	178 (39.38)	274 (60.62)
Type 3	543 (27.89)	177 (32.60)	366 (67.40)
Type 4	338 (17.36)	107 (31.66)	231 (68.34)
Type 5	280 (14.38)	103 (36.79)	177 (63.21)
BI-RADS breast density			
Type 1	26 (2.05)	N.A	26 (2.05)
Type 2	282 (22.26)	N.A	282 (22.26)
Type 3	730 (57.62)	N.A	730 (57.62)
Type 4	229 (18.07)	N.A	229 (18.07)
Age at menarche (years)			
7–11	72 (3.70)	28 (38.89)	44 (61.11)
12–13	682 (35.03)	315 (46.19)	367 (53.81)
≥ 14	1193 (61.27)	337 (28.25)	856 (71.75)
Number of pregnancy			
0	94 (4.85)	67 (71.28)	27 (28.72)
1	454 (23.43)	226 (49.78)	228 (50.22)
2	578 (29.82)	177 (30.62)	401 (69.38)
3–4	664 (34.26)	169 (25.45)	495 (74.55)
≥ 5	148 (7.64)	34 (22.97)	114 (77.03)
Missing	9	7	2

The mean age of participants was 45.40 ± 9.77 years. 680 women were 30–39 years old and 1,267 were 40–69 years old (Table 1). Generally, the study included 786 BI-RADS lesions class 1–2 (40.37%), 543 BI-RADS lesions class 3 (27.89%), 338 BI-RADS lesions class 4 (17.36%) and 280 BI-RADS lesions class 5 (14.38%).

### Cancer detection

In the age group of 30–39 (680 subjects), HHUS detected 79 cases of breast cancer (11.62%), including 70 cases of invasive carcinoma (10.29%) and 9 cases of non-invasive carcinoma (1.32%). ABUS detected 75 cases of breast cancer (11.03%), including 65 cases of invasive carcinoma (9.56%) and 10 cases of non-invasive carcinoma (1.47%). (Table 2) In the 30–39 age group, the mean diameter of cancer detected by HHUS was 22.74 ± 11.05 mm, and the average diameter of cancer detected by ABUS was 19.78 ± 10.83 mm (Table 3).

**Table 2:** Cancer Detection of HHUS, ABUS and MG.

	30–39 years age group (n = 680)			40–69 years age group(n = 1267)					All Participants(n = 1947)		
	Non-invasive	Invasive	Sum	Non-invasive		Invasive		Sum	Non-invasive	Invasive	Total
				LD	HD	LD	HD				
HHUS (%)	9 (1.32%)	70 (10.29%)	79 (11.62%)	7 (0.55%)	21 (1.66%)	89 (7.02%)	197 (15.55%)	314 (24.78%)	37 (1.90%)	356 (18.28%)	393 (20.18%)
ABUS (%)	10 (1.47%)	65 (9.56%)	75 (11.03%)	9 (0.71%)	21 (1.66%)	86 (6.79%)	190 (15.00%)	306 (24.15%)	40 (2.05%)	341 (17.51%)	381 (19.57%)
MG (%)	N/A	N/A	N/A	6 (0.47%)	19 (1.50%)	86 (6.79%)	172 (13.58%)	283 (22.34%)	25 (1.28%)	258 (13.25%)	283 (14.54%)
Pathology (%)	15 (2.21%)	71 (10.44%)	86 (12.65%)	11 (0.87%)	26 (2.05%)	90 (7.10%)	202 (15.95%)	329 (25.97%)	52 (2.67%)	363 (18.64%)	415 (21.31%)

LD: lower-Low-density breast subgroup; HD: High-density breast subgroup; SUM: summation.

**Table 3:** Cancer size distribution.

	Cancer size in 30–39 years age group			Cancer size in 40–69 years age group					Cancer size in All participants		
	Non-invasive	Invasive	Sum	Non-invasive		Invasive		Sum	Non-invasive	Invasive	Total
				LD	HD	LD	HD				
HHUS Mean (SD)	22.87 (12.45)	22.72 (10.83)	22.74 (11.05)	19.55 (11.47)	24.69 (13.66)	21.61 (9.00)	23.29 (10.74)	22.82 (10.59)	23.08 (12.80)	22.76 (10.35)	22.80 (10.68)
ABUS Mean (SD)	19.67 (15.14)	19.81 (9.79)	19.78 (10.83)	15.18 (8.51)	19.64 (14.06)	19.02 (8.56)	20.83 (10.30)	20.04 (10.12)	18.62 (13.25)	20.17 (9.79)	19.98 (10.26)
MG Mean (SD)	N/A	N/A	N/A	19.00 (11.66)	19.20 (9.40)	21.26 (6.49)	22.58 (9.67)	21.87 (8.70)	19.13 (9.78)	22.08 (8.60)	21.87 (8.70)

LD: lower-Low-density breast subgroup; HD: High-density breast subgroup; SUM: summation.

In the 40–69 age group (1,267 subjects) (Table 2), HHUS detected a total of 314 breast cancers (24.78%), of which 286 (22.57%) were invasive and 28 (2.21%) were non-invasive. 197 cases of invasive carcinoma (15.55%) were detected in the high-density subgroup and 89 cases of invasive carcinoma (7.02%) in the low-density subgroup. ABUS detected 306 cases of breast cancer (24.15%), including 276 cases of invasive carcinoma (21.79%) and 30 cases of non-invasive carcinoma (2.37%). 190 cases of invasive carcinoma (15.00%) were detected by ABUS in the high-density subgroup and 86 cases of invasive carcinoma (6.79%) by the low-density subgroup. MG detected 283 cases of breast cancer (22.34%), including 258 cases of invasive carcinoma (20.37%) and 25 cases of non-invasive carcinoma (1.97%). In the high density subgroup, MG detected 172 invasive cancers (13.58%). In the low-density subgroup, MG detected 86 invasive cancers (6.79%). In the 40–69 age group, the mean diameter of cancer detected by HHUS was 22.82 ± 10.59 mm, the mean diameter of cancer detected by ABUS was 20.04 ± 10.12 mm, and the mean diameter of cancer detected by MG was 21.87 ± 8.70 mm (Table 3).

In total (1947 subjects) (Table 3), 415 (21.31%) were confirmed by final pathology, 363 (18.64%) were invasive, and 52 (2.67%) were non-invasive. HHUS detected 393 cases of breast cancer (20.18%), including 356 cases of invasive carcinoma (18.28%) and 37 cases of non-invasive carcinoma (1.90%). ABUS detected 381 breast cancers (19.57%), of which 341 were invasive (17.51%) and non-invasive 40 (2.05%). MG detected 283 cases of cancer (14.54%), including 258 cases of invasive carcinoma (13.25%), non-invasive carcinoma in 25 cases (1.28%). The tumor diameters of the 30–39 years group and the 40–69 years age group were combined to calculate. The average diameter of cancer detected by HHUS was 22.80 ± 10.68 mm, and the average diameter of cancer detected by ABUS was 19.99 ± 10.26 mm.

### Non-inferiority and superiority analysis of sensitivity

#### ABUS vs. HHUS

In 30–39 age group (Table 4), non-inferior Z tests showed that ABUS sensitivity (87.21%) was non-inferior to HHUS sensitivity (91.86%) with *P* = 0.325. As HHUS sensitivity was higher than the sensitivity of ABUS, it lead to superiority test of ABUS vs. HHUS was not available.

**Table 4:** Non-inferiority and superiority analysis results in 40–69 years age group.

	Low-density subgroup					High-density subgroup					40–69 years group				
	Sensitivity% (95% CI)	Specificity% (95% CI)	FPR% (95% CI)	AC		Sensitivity% (95% CI)	Specificity% (95% CI)	FPR% (95% CI)	AC		Sensitivity %(95%CI)	Specificity%(95%CI)	FPR% (95%CI)	AC	
HHUS	95.05% (88.93, 97.87)	92.27% (87.81, 95.19)	7.73% (4.81 12.19)	93.18%		95.61% (92.12, 97.60)	88.78% (86.29, 90.87)	11.22% (9.13, 13.71)	90.41%		95.44% (92.61, 97.22)	89.55% (87.43, 91.35)	10.45% (8.65, 12.57)	91.08%	
ABUS	94.06% (87.64, 97.25)	94.69% (90.74, 97.01)	5.31% (2.99, 9.26)	94.48%		92.54% (88.38, 95.29)	92.34% (90.18, 94.05)	7.66% (5.95, 9.82)	92.39%		93.01% (89.73, 95.3)	92.86% (91.03, 94.34)	7.14% (5.66, 8.97)	92.9%	
MG	91.09% (83.93, 95.24)	95.65% (91.95, 97.70)	4.35% (2.30, 8.05)	94.16%		83.77% (78.43, 87.99)	90.56% (88.22, 92.47)	9.44% (7.53, 11.78)	88.95%		86.02% (81.85, 89.35)	91.68% (89.74, 93.29)	8.32% (6.71, 10.26)	90.21%	
P*_A VS. H_	0.051	< 0.001				0.061	< 0.001				0.014	< 0.001			
P*_A VS. M_	0.008	0.007				< 0.001	< 0.001				< 0.001	< 0.001			
P*_H VS. M_	0.002	0.088				< 0.001	0.001				< 0.001	< 0.001			
*P*^*#*^_*A VS. H*_	0.353	0.029				0.035	< 0.001				0.044	< 0.001			
*P*^*#*^_*A VS. M*_	0.183	NA				0.002	0.061				0.001	0.114			
*P*^*#*^_*H VS. M*_	0.079	NA				< 0.001	NA				< 0.001	NA			

*Non-inferiority *P* value; # superiority *P* value; FPR: false positive rate; AUC: area under curve; AC: Accuracy.

In the 40–69 age group (Table 4), non-inferior Z tests showed that ABUS sensitivity (93.01%) was non-inferior to HHUS sensitivity (95.44%) with *P* = 0.014. Superiority test of HHUS vs. ABUS is also not available.

For all participants (Table 5), ABUS sensitivity (91.81%) compared with HHUS sensitivity (94.70%) with non-inferior Z tests, *P* = 0.015. Therefore, it can be inferred that the overall sensitivity of ABUS are not inferior to that of HHUS. Superiority test of HHUS vs. ABUS for all participants is not available.

**Table 5:** General non-inferiority and superiority study between HHUS and ABUS.

	30–39 years age group				40–69 years age group					All participants			
	Sensitivity% (95% CI)	Specificity% (95% CI)	FPR% (95% CI)	AC	Sensitivity% (95% CI)		Specificity% (95% CI)	FPR% (95% CI)	AC	Sensitivity % (95%CI)	Specificity % (95%CI)	FPR % (95%CI)	AC
HHUS	91.86% (84.14, 96.00)	89.06% (86.29, 91.32)	10.94% (8.68, 13.71)	89.41%	95.44% (92.61, 97.22)		89.55% (87.43, 91.35)	10.45% (8.65, 12.57)	91.08%	94.7% (92.10, 96.47)	89.36% (87.72, 90.81)	10.64% (9.19, 12.28)	90.5%
ABUS	87.21% (78.53, 92.71)	92.93% (90.58, 94.73)	7.07% (5.27, 9.42)	92.21%	93.01% (89.73, 95.30)		92.86% (91.03, 94.34)	7.14% (5.66, 8.97)	92.90%	91.81% (88.77, 94.08)	92.89% (91.49, 94.07)	7.11% (5.93, 8.51)	92.66%
P*_A VS. H_	0.325	< 0.001			0.014		< 0.001			0.015	< 0.001		
*P*^*#*^_*A VS. H*_	NA	< 0.001			NA		< 0.001			N/A	< 0.001		

*Non-inferiority *P* value; ^#^superiority *P* value; FPR: false positive rate; AUC: area under curve; AC: Accuracy.

#### ABUS/HHUS vs. MG

In the 40–69 age group (Table 4), non-inferior Z tests showed that ABUS sensitivity (93.01%) was non-inferior to MG sensitivity (86.02%) with P < 0.001 and HHUS sensitivity (95.44%) was non-inferior to MG sensitivity (86.02%) with P < 0.001. Sensitivity of ABUS and HHUS are all superior to MG with P < 0.001 by superior test.

In high-density breast subgroup (Table 4), non-inferior Z tests showed that ABUS sensitivity (92.54%) was non-inferior to MG sensitivity (83.77%) with *P* < 0.001 and HHUS (95.61%) sensitivity was non-inferior to MG sensitivity (83.77%) with *P* < 0.001. Superiority Mcnemar test show that ABUS sensitivity was superior to MG sensitivity, *P* = 0.002 and HHUS sensitivity was superior to MG sensitivity, *P* < 0.001.

In low-density breast subgroup (Table 4), non-inferior Z tests showed that ABUS sensitivity (94.06%) was non-inferior to MG sensitivity (91.09%) with *P* = 0.008 and HHUS (95.05%) sensitivity was non-inferior to MG sensitivity (91.09%) with *P* < 0.001. Superiority Mcnemar test show that ABUS sensitivity was not superior to MG sensitivity, P = 0.183 and HHUS sensitivity was not superior to MG sensitivity, *P* = 0.079.

### Non-inferiority and superiority analysis of specificity

#### ABUS vs. HHUS

In 30–39 age group (Table 4), non-inferior Z tests showed that ABUS specificity (92.93%) was non-inferior to HHUS specificity (89.06%) with *P* < 0.001. Superiority Mcnemar test show that ABUS specificity was superior to HHUS specificity, *P* < 0.001.

In the 40–69 age group (Table 4), non-inferior Z tests showed that ABUS specificity (92.86%) was non-inferior to HHUS specificity (89.55%) with *P* < 0.001. Superiority test show that ABUS specificity was superior to HHUS specificity, *P* < 0.001.

For all participants (Table 5), ABUS specificity (92.89%) was non-inferior to HHUS specificity (89.36%) with *P* < 0.001. Superiority test show that specificity of ABUS was superior to that of HHUS with *P* < 0.001.

#### ABUS/HHUS vs. MG

In the 40–69 age group (Table 4), non-inferior Z tests showed that ABUS specificity (92.86%) was non-inferior to MG specificity (91.68%) with *P* < 0.001 and HHUS specificity (89.55%) was non-inferior to MG specificity (91.68%) with *P* < 0.001. ABUS is not superior to MG with P = 0.114 by superior test. Superiority of HHUS vs. MG is not available.

In high-density breast subgroup (Table 4), non-inferior Z tests showed that ABUS specificity (92.34%) was non-inferior to MG specificity (90.56%) with P < 0.001 and HHUS (88.78%) specificity was non-inferior to MG specificity (90.56%) with P < 0.001. Superiority Mcnemar test show that ABUS specificity was not superior to MG specificity, P = 0.061. Superiority test of HHUS vs. MG was not available.

In low-density breast subgroup (Table 4), non-inferior Z tests showed that ABUS specificity (94.69%) was non-inferior to MG specificity (95.65%) with *P* = 0.007 and HHUS (92.27%) specificity was non-inferior to MG specificity (95.65%) with *P* < 0.001. Superiority Mcnemar test was not available.

## Discussion

This multicenter study demonstrated that ultrasound (ABUS or US) is superior to mammography in dense breast patients, but perform as good as X-rays in low-dense breast patients. Furthermore, we found that both ABUS and HHUS, the sensitivity is superior to MG, ABUS specificity (92.34%) was non-inferior to MG specificity (90.56%). This conclusion suggests that US at least in symptomatic populations is more effective at detecting breast cancer than MG.

**Figure 2:**
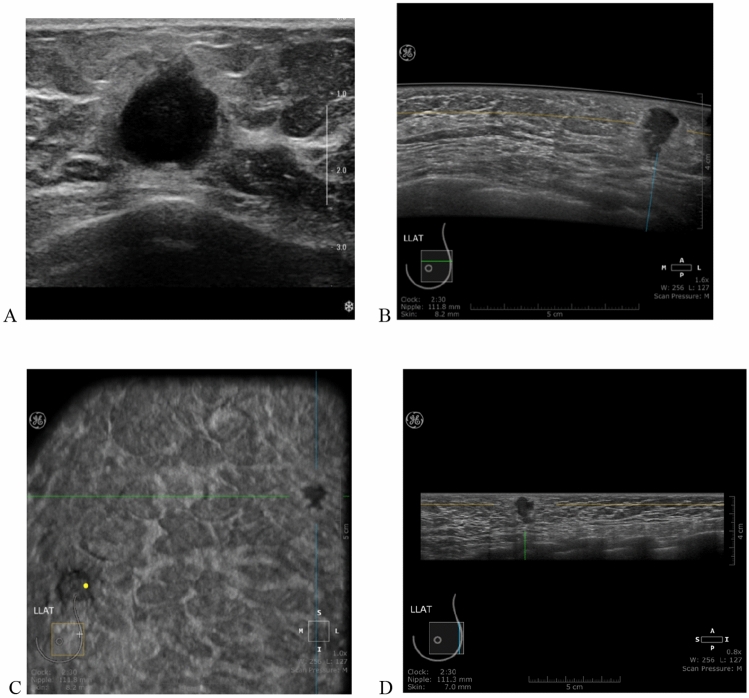
A 32-year-old woman with ductal carcinoma of the left breast. (**A**) shows a hypoechoic lesion with handheld ultrasound (HHUS). And (**B**,**C**,**D)** show the heterogeneous hypoechoic lesions in the medial (**B**), lateral (**D**), and anterior–posterior (**C**) position of Automated Breast Ultrasound (ABUS).

ABUS have been shown to achieve the same diagnostic accuracy as HHUS^18,28,29^. In Su Kyung Jeh study, the diagnostic performance of ABUS was higher than that of HHUS in respect of specificity and accuracy^29^. Chang et al.^25^ reported that both ABUS and HHUS had high sensitivity (both 100%) and high specificity (95.0% and 85.0%, respectively) for 69 lesions. In addition, the ABUS had a higher diagnostic accuracy (97.1%) than HHUS (91.4%) for breast masses. The authors concluded that ABUS is a promising modality in breast imaging. In our study, ABUS achieved higher accuracy than HHUS (ABUS 92.66% vs. HHUS 90.50% in all subjects; ABUS 92.90% vs. HHUS 91.08% in the 40–69 age group). In addition, ABUS had the highest specificity compared to HHUS, ABUS and MG (ABUS 92.89% vs. HHUS 89.36% in all subjects; ABUS 92.86% vs. MG 91.68% vs. HHUS 89.55% in the 40–69 age group) .This may be because ABUS can display more coronal plane-related information such as mass margins, shape, spiculations, and distortion associated with tissue retraction (Fig. 2). Meanwhile, breast cancer detection rates were higher in HHUS and ABUS than in MG (20.18% of HHUS / 19.57% of ABUS vs. 14.54% of MG). Most of the breast cancers detected are invasive breast cancer. The reason maybe that most of the participants were actually symptomatic and had tumor diameters with mean diameter close to 20 mm.

In the study, the sensitivity of ABUS was lower than that of HHUS, which may be related to the compression of mammary gland tissue during the operation of ABUS, resulting in unclear display of some lesions.However, from the non-inferiority analysis results, sensitivity of ABUS is not inferior to HHUS. Meanwhile, specificity of ABUS is superior to HHUS in both the 30–39 age group and the 40–69 age group. This may be due to the additional information of the coronal plane and it helps to differentiate between benign and malignant breast lesions^18,30–32^. Therefore, on the issue of diagnostic efficacy, ABUS and HHUS have their own strengths and weaknesses in sensitivity and specificity. Moreover, non-inferiority tests also demonstrated that US (ABUS/HHUS) specificity is non-inferior to MG. Meanwhile, it must also be noted that the ABUS diagnosis is actually made by a radiologist with considerable HHUS experience. In addition, according to the ABUS user interface and habits of the process, operators are generally the first to read the conventional 2-D information before interpretation of the coronal plane. Therefore, the diagnostic decision of ABUS may come from the diagnostic information of the conventional 2-D section more in routine ABUS operation. Thus, the comparison of the diagnostic efficacy between ABUS and HHUS may reflect, to a greater extent, the capability comparison between two experienced HHUS radiologists, other than ability contrast of two different machines. However, the greater strength of ABUS lies in its standardized cross-section and its potential tele-consultation capabilities, which empower experienced radiologist to work for less-experienced areas.

The distinction between efficacy as measured in experimental studies and the effectiveness of a mass population intervention is a crucial one for public health decision-making. Therefore, the limitation of this study is that all the conclusions come from the symptomatic population, thus limiting the extension of evidence to the asymptomatic population. In the future, there is still a need for randomized controlled validation study in asymptomatic populations.

In summary, the sensitivity of ABUS/HHUS is superior to that of MG. The specificity of ABUS/HHUS is non-inferior to that of MG. Therefore, given the affordability, feasibility and good performance of ultrasound, ABUS provides a standardized and reproducible imaging device that can be used for breast cancer detection. Our study suggests that large-scale, multicenter randomized controlled studies are warranted to confirm the benefits of breast cancer screening by ABUS.
